# RNA Loading on Nano-Structured Hyperbranched β-Cyclodextrin

**Published:** 2018

**Authors:** Sorina Hirbod, Shohreh Nafisi, Howard I Maibach

**Affiliations:** 1. Department of Chemistry, Central Tehran Branch, Islamic Azad University, Tehran, Iran; 2. Department of Dermatology, University of California, San Francisco, CA, USA

**Keywords:** Aggregation, Gene delivery, Particle size, Polymer

## Abstract

**Background:**

β-Cyclodextrin functionalized hyper-branched polyglycerol (HBCD: β-CD-g-PG), a biocompatible polymer, has recently been proposed for delivery of poorly water soluble compounds.

**Methods:**

The present study examines the interaction of HBCD with RNA, utilizing a constant concentration of RNA and different HBCD/RNA ratios of 1/16 to 1/1, at physiological condition in an aqueous solution. Circular Dichroism (CD), UV-visible, FTIR spectroscopic methods, zeta potential and Dynamic Light Scattering (DLS) were used to analyze the particle formation, particle charge, particle size, aggregation, RNA conformation, binding constant and mode, and the effect of polymer complexation on RNA stability.

**Results:**

The results indicate that the interaction of RNA with HBCD leads to the formation of a linear dendritic supramolecule biopolymer with an overall binding constant of K_HBCD/RNA_= 1.25 × 10^3^.

**Conclusion:**

The small sized synthesized polymer can be considered as an appropriate system for preventing RNA aggregation and protecting the gene by host-guest interaction.

## Introduction

Recently, a variety of macromolecule structures such as Cyclodextrins (CDs) and dendrimers have been examined for the formation of stable complexes with genes^1–6^. CDs, cyclic oligosaccharides are well-known structures in supramolecular polymers in which the monomers are held together by reversible and highly directional non-covalent bonds ^7,8^. These biocompatible and nontoxic compounds ^9^ possess a cage-like supramolecular structure, with hydrophilic outer surfaces and lipophilic inner cavities. Various hydrophobic drugs can be embedded in their hydrophobic cavities *via* non-covalent interactions ^10–14^. However, CDs can only cause a minor enhancement in the solubility of hydrophobic drugs ^15,16^.

Recent advances have been focused on functionalizing CDs to obtain small sized macromolecules with higher free cavities and water solubility ^17–19^. Polyglycerol dendron functionalized cyclodextrins (β-CD-g-PG) are novel carriers which can be synthesized by controlled anionic polymerization of glycidol. The Hyperbranched Cyclodextrins (HBCD) are small sized, well-defined polymers with higher water solubility, oxidative stability and biocompability. They exhibit high potential of encapsulating hydrophobic guest molecules due to the molecules binding to interior or exterior part of their structure ^20–22^.

Tao *et al* studied the host-guest interaction between β-CD-g-PG with long alkyl chain adamantines ^23^. The incorporation of insulin into HBCD polymers significantly caused enhanced drug absorbance across the nasal barrier and decreased blood glucose concentration ^24^. Paclitaxel was effectively encapsulated by HBCD for targeted drug delivery ^25^. Recently, β-cyclodextrine-polyethyleneimine polymers (PEI-CDs) were examined for *in vitro* transport of miRNA(microRNA) and siRNA (small interfering RNA) ^6,26^. CDs have also been studied as topical drug delivery systems. Application of CDs derivatives in transdermal drug delivery has improved drug release/permeation, decreased druginduced local irritationand hasoptimized systemic and local dermal drug delivery. Recently, Loftsson *et al* reviewed the role of cyclodextrins on drug delivery through biological membranes ^27^.

Although several experiments report the interaction of CDs with guest molecules ^28,29^, there is no precise information on the molecular aspects of interaction between HBCD and RNA in aqueous solution. Thus, HBCD interaction with RNA was studied in aqueous solutions with HBCD/RNA molar ratios of 1/16 to1/1 at pH=6.5–7.5 utilizing CD, UV, and FTIR measurements. The complexes were analyzed to study the particles size and charge parameters. The structural analyses of RNA secondary structures, binding site of HBCD to RNA, and HBCD-RNA binding constant were provided by spectroscopic results.

## Materials and Methods

### Materials

Sodium methoxide, methanol and acetone were purchased from Merck. β-cyclodextrin, glycidol and Baker’syeast RNA from Sigma Chemical (St. Louis, MO) were employed without further purification. The absorbance band at 280 *nm* was utilized to check the protein content of RNA solutions. The A260/A280 ratio for RNA was 2.10, indicating that the RNA samples were nearly free from protein ^30^. Other chemicals were of reagent grade and used without further purification.

### Synthesis of HBCD polymer

HBCD (β-CD-g-PG) was synthesized by anionic ring opening multi branching polymerization method ^20^. Briefly, β-CD (0.5 *g*) was deprotonated by dissolving sodium methoxide (14.4 *mmol*) in dried methanol. Mixture was stirred at room temperature using a polymerization ampule equipped with a magnetic stirrer for 1 *hr*. Methanol was vaporized *via* vacuum oven at 60°*C* for 2 *hr*. Glycidol (92 *mmol*) was added to deprotonated β-CD and temperature was gradually increased up to 120°*C*. The mixture was stirred at 120°*C* for 12 *hr* and allowed to cool. It was dissolved in pure methanol, and precipitated upon addition of aceton. It was neutralized by filtration over cation-exchange resin. The precipitate was dried *via* vacuum oven at 80°*C*. Finally, a yellow viscous product was obtained with 87% yield.

### Preparation of stock solutions

Homogeneous sodium-RNA solutions (1% w/w: 10 *mg/ml*) were prepared by dissolving RNA in 10 *ml* of phosphate buffer (pH=7.4) with occasional stirring at 5°*C* for 24 *hr*. Using the molar extinction coefficient of 6600 *cm*^−1^ *M*^−1^ which was represented as molarity of phosphate groups, the final concentration of RNA stock solution was assessed spectrophotometrically at 260 *nm* ^31,32^. UV absorbance of the diluted RNA solution (40 *μM*) at 260 *nm* was 0.11 (with the path length of 1 *cm*), and the final concentration of RNA stock solution was 25 *mM* in phosphate. For infrared spectroscopic measurements, various amounts of HBCD in phosphate buffer (pH=7.4) were added dropwise to RNA solutions to achieve favorable HBCD contents of 0.625, 1.25, 2.5, 5 and 10 *mM* with an ultimate RNA concentration of 10 *mM*. Infrared spectra were recorded 1 *hr* after the mixing of HBCD solutions with RNA solutions. For UV measurements, various concentrations of HBCD (500–1200 *μM*) were used with constant RNA concentration (40 *μM*).

**Table 1.** Zeta potential of RNA, HBCD and RNA-HBCD

**Sample [(Concentration (*μM*)]**	**ζ(*mV*)**
**RNA (40** ***μM*)**	−8.47
**HBCD (1000** ***μM*)**	−1.8
**HBCD-RNA**	−9.22

### FTIR spectroscopy measurements

To record the infrared spectra, a Nicolet FTIR spectrometer (Magna 550) equipped with a liquid-nitrogencooled HgCdTe (MCT) detector was used. The spectra of the HBCD/RNA solutions were collected utilizing a cell assembled with ZnSe windows and treated by OMNIC software. The spectra of solutions were recorded after 1 *hr*. The bands were measured in three individual samples, at the same HBCD and RNA concentrations. 100 scans were collected for each spectrum with resolution of 4 *cm*^−1^. To obtain difference spectra [(polynucleotide solution +HBCD solution)-(polynucleotide solution)], a sharp RNA band at 968 *cm*^−1^ was used as internal reference ^33,34^. This band is assigned to sugar C-C and C-O stretching vibration and does not change (intensity or shifting variation) upon HBCD complexation to RNA, so it will be deleted upon spectral subtraction.

**Figure 1.**
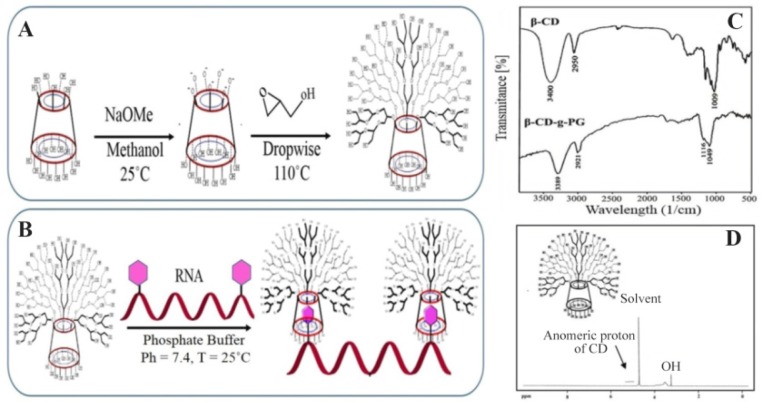
A) HBCD synthesis by anionic ring opening polymerizations. B) HBCD-RNA synthesis by host-gust interactions between cavity of HBCD polymers and RNA bases. C) FTIR spectra of β-CD and HBCD. D) ^1^H-NMR spectra of HBCD in the D_2_O as solvent.

By considering the reference band at 968 *cm*^−1^ (RNA) as a function of HBCD concentrations, the intensity ratios of RNA in-plane vibrations related to phosphate stretching vibrations and A-U and G-C base pairs were measured with an error of ±0.9%. In order to determine the ligand binding to RNA bases and backbone phosphate groups, similar intensity variations have been used ^35,36^. After peak normalization, the plot of relative intensity (R) of various RNA in-plane vibrations including 1698 (guanine), 1650 (uracil), 1606 (adenine), 1489 (cytosine) and 1241 *cm*^−1^ (phosphate groups) versus polymer concentrations were gained. The peak normalization was performed using R_i_ = I_i_/I_968_ where I_i_ is the intensity of the absorption peak for pure RNA in the complex with i as ligand concentration, and I_968_ as the intensity of the peak at 968 *cm*^−1^ (RNA internal reference) (Figure 1).

### Absorption spectroscopy

A LKB model 4054 UV-Visible spectrometer was used to record the absorption spectra; quartz cuvettes of 1 cm were used with various HBCD concentrations of 5×10^−4^–12×10^−4^ *M* and constant RNA concentration of 40×10^−6^ *M*. Binding constants of HBCD-RNA complexes were calculated as reported ^37^. The interaction between HBCD as ligand [L] and RNA as substrate [S] is assumed to be 1:1 which forms a single complex SL (1:1). Equation (1) describes the relationship between the system variables and parameters with the observed absorbance change per centimeter:

(1) $$\frac{\Delta A}{b}=\frac{S_{t}K_{11}\Delta \varepsilon_{11}[L]}{1+K_{11}[L]}$$

ΔA=A−A_0_ from the mass balance expression St=[S]+ [SL], [S]=St/(1+K_11_[L]) is obtained

Equation (1) is the binding isotherm, which indicates the hyperbolic dependence on free ligand concentration. According to the following equation, the linearization of equation (1) yields the double-reciprocal form of plotting the rectangular $\frac{1}{y}=\frac{f}{d}\cdot \frac{1}{x}+\frac{e}{d}$ hyperbola:

(2) $$\frac{b}{\Delta A}=\frac{1}{S_{t}K_{11}\Delta \varepsilon_{11}[L]}+\frac{1}{S_{t}\Delta \varepsilon_{11}}$$

As the double reciprocal plot of 1/ΔA versus 1/[L] is linear, the binding constant can be estimated from the following equation:

(3) $$K_{11}=\frac{\text{intercept}}{\text{slope}}$$

### CD spectroscopy

Various HBCD concentrations (125, 250, 500, 1000 and 2000 *μM*) were mixed with constant concentration of RNA (40 *μM*). The spectra of free baker’s yeast RNA and HBCD-RNA adducts were recorded using a Jasco J-720 spectropolarimeter at pH=7.3. A quartz cell with a path length of 1 *cm* was used for far-UV region (200–260 *nm*) measurements. Six scans were accumulated at a scan speed of 50 *nm/min*, with data being collected at every nanometer from 200 to 260 *nm*. The temperature of sample was kept at 25°*C* using a Neslab RTE-111 circulating water bath connected to the water-jacketed quartz cuvette. Using Jasco Standard Analysis software, spectra were corrected for buffer signal, and a conversion to Mol CD (Δε) was performed.

### Determination of size and potential

To determine polydispersity and the average diameter of HBCD-RNA polymer, free HBCD (1000 *μM*) and free RNA (40 *μM*), a Dynamic Light Scattering (DLS) particle size analyzer (Brookhaven-model: 90plus USA) was used. A zeta potential meter (Brookhaven-model: ZetaPALS) was used to measure zeta potential of HBCD-RNA complexes, free HBCD (1000 *μM*) and free RNA (40 *μM*) at 37°*C*.

## Results

### IR and ^1^H-NMR of HBCD

**Figure 2.**
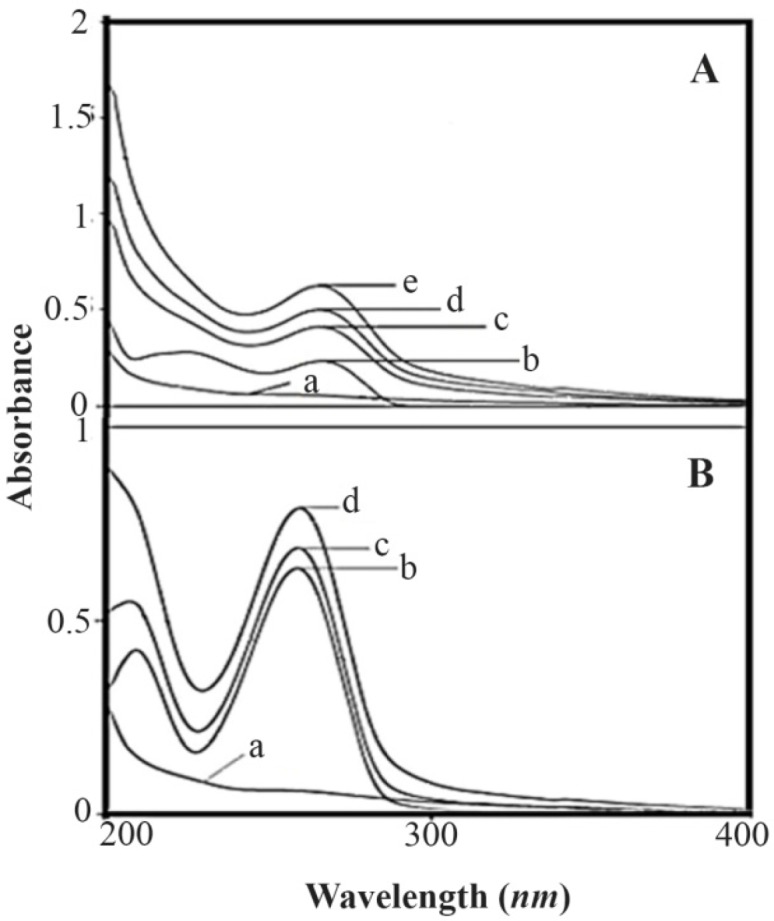
A) UV-visible spectra of (a) HBCD (100 *μM*); (b) RNA (40 *μM*), (cj) HBCD-RNA complexes: (c) 250, (d) 300, (e) 350, (f) 400, (g) 450, (h) 500, (i) 550, (j) 600 *μM*. B) Plot of 1/(A–A0) *vs.* (1/polymer concentration) for HBCD and RNA complexes, where A0 is the initial absorbance of RNA (260 *nm*) and A is the recorded absorbance (260 *nm*) at different polymer concentrations (250–600 *μM*) with constant RNA concentration of 40 *μM* at pH=7.4.

HBCD polymer was synthesized by anionic ring opening polymerization method (Figure 2A) which was previously reported ^20,28^. The IR spectral changes of β-CD and HBCD are presented in figure 2B.

Absorption bands at 3380, 1116 and 2921 *cm*^−1^ are related to O-H, C-O-C and C-H groups, respectively ^20,28^ . Broadening of the C-O band in HBCD spectrum comparing with β-CD can be assigned to polyglycerol binding to cyclodextrin functional groups. Similar broadening in the C-O absorbance of HBCD compared with β-CD was assigned to polyglycerol binding to the OH groups of cyclodextrin ^20,28^.

In the HBCD^1^H NMR spectrum (Figure 2C), the signals of cyclodextrin protons have overlapped with polyglycerol signals at 4.1–3.6 *ppm*. The weak signal at 5.2 *ppm* is related to anomeric protons of cyclodextrin. The same ^1^H-NMR results for HBCD was reported by Adeli *et al* ^28,38^.

### Infrared spectra of HBCD-RNA complexes

FTIR spectral studies have identified weak interaction of HBCD with phosphate and bases of RNA at low HBCD concentrations (*via* host-guest) and major participation of bases mainly with guanine, cytosine and backbone phosphate groove at higher concentrations. Evidence for this comes from the spectral changes of free RNA upon HBCD complexation.

**Figure 3.**
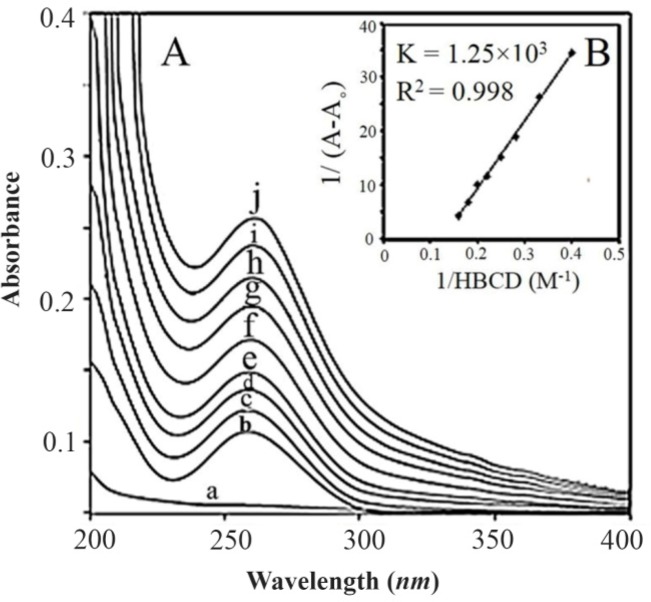
FTIR spectra in the region of 1800–600 *cm*^−1^ for (A) Baker’s yeast RNA (10 *mM*) (B) HBCD (C) HBCD-RNA: RNA (10 *mM*), HBCD (5 *mM*) (D) HBCD-RNA: RNA (10 *mM*), HBCD (10 *mM*) in aqueous solution at pH=7. RNA, HBCD and two complexes at various HBCD/RNA (phosphate) molar ratios (1/2, 1/1) (four top spectra); two difference spectra (bottom two spectra).

At low HBCD concentration (r=1/16 to 1/8), no significant interaction was observed between HBCD and RNA bases as represented by small shifting and intensity changes of RNA in-plane vibrations at 1698 (G, U mainly G), 1650 (U, G, A, C mainly U), 1606 (A), 1489 (C) and 1241 *cm*^−1^ (asymmetric PO_2_ stretch) (Figures 1 and 3). The difference spectra showed negative features at 1724 and 1782 *cm*^−1^ which is due to the loss of RNA intensity vibrations (Figure 1). This is indicative of a minor, indirect interaction of RNA with HBCD *via* H-bonding system at low HBCD concentrations. Similarly, the loss of DNA vibrations intensity and negative features at cisplatin-DNA spectrum was assigned to minor and indirect H-bonding interaction of DNA with CD-37 ^39^.

At higher HBCD concentrations (r=1/4), major shifting of the guanine and cytosine bands (G band at 1698 to 1680 *cm*^−1^ (1/1); C band at 1489 *cm*^−1^ to 1474 (r=1/4) was observed and the intensities of the adenine, guanine, cytosine and uracil bands strongly augmented. The intensity increase was characterizedby the presence of several positive features at 1715 (guanine), 1650 (uracil), 1510, 1490 *cm*^−1^(cytosine) and 1245 (phosphate) in the difference spectrum of HBCD-RNA (Figures 1 and 3; the difference spectrum is not shown in figure 1). The spectral changes (intensity and shifting) for the A, G, C and U bases (r=1/4) can be assigned to the interaction of HBCD (through OH) with mainly guanine and cytosine and to a lesser extent with the adenine and uracil bases.

At higher HBCD concentrations (r=1/2, 1/1), drastic shifting of the guanine and cytosine bands (guanine band at 1698 to 1706 *cm*^−1^ (1/1); cytosine band at 1489 to 1460 (r=1/2), 1477 *cm*^−1^ (r=1/1)) were observed. The spectral changes for the bases (r=1/2 and 1/1) can be attributed to the interaction of OH branches of HBCD with mainly G and C and to a lesser extent with A and U bases (Figures 1 and 3). Similar spectral changes were observed in narigin-RNA complexes ^40^.

HBCD-PO2 interaction was evident from intensity increase and shifting of the PO2 antisymmetric band at 1241 and symmetric band at 1085 *cm*^−1^ in the spectra of HBCD-RNA complexes (Figures 1 and 3).

For the phosphate group, shifting of the band at 1241 *cm*^−1^ to 1243 (r=1/8), 1245 (r=1/4), 1253 (r=1/2) and 1249 *cm*^−1^ (r=1/1) for the complexes can be ascribed to minor phosphate interaction with HBCD at lower concentrations and strong interaction at higher concentrations (r=1/2, 1/1). Similar spectral changes were observed in PAMAM-tRNA complexes ^36^.

### Circular dichroism spectra and RNA conformation

**Figure 6.**
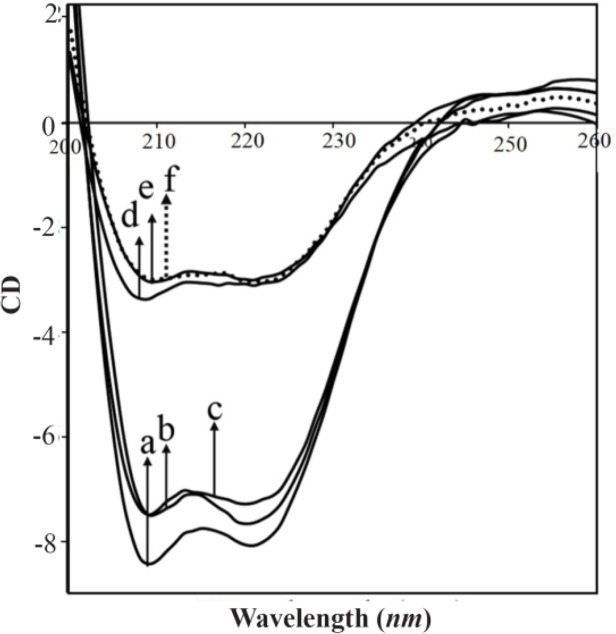
DLS diagrams (phosphate buffer, pH=7.4 at 37°*C*); A) RNA (40 *μM*), B) HBCD (1000 *μM*) C) HBCD-RNA complexes.

CD spectra of RNA and its complexes with different HBCD concentrations are shown in figure 6. CD spectrum of RNA, composed of four major peaks at 209 (negative), 221 (negative), 240 (negative), and 269 *nm* (positive) (Figure 3) is consistent with previously reported CD spectra of double-helical RNA in a conformation ^38,41^.

**Figure 4.**
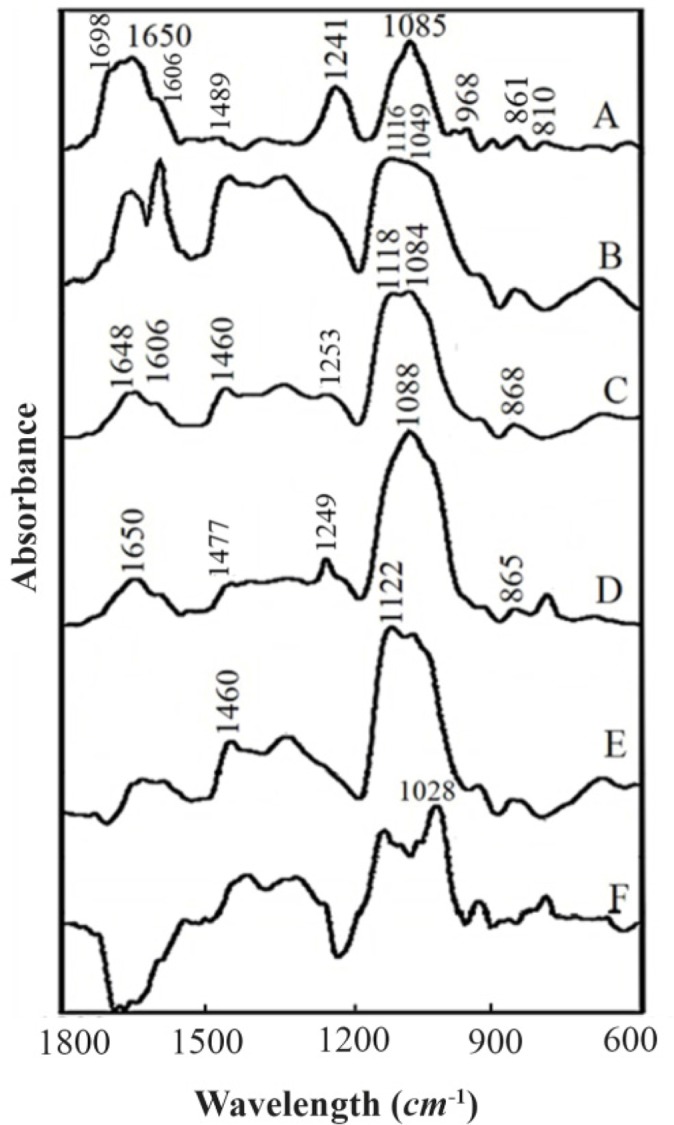
Intensity ratio variations for various RNA in-plane vibrations as a function of HBCD concentration. Intensity ratios for RNA bands at 1698 (G, U), 1650 (U,G,A,C), 1606 (A), 1489 (C,G) and 1241 (PO2 asymmetric) referenced to RNA band at 969 *cm*^−1^.

At low HBCD concentration (125, 250 *μM*), no significant shifting was observed; however, the molar ellipticity of the band at 209 *nm* was strongly enhanced by increasing the polymer concentration (500 to 2000 *μM*). In the spectra of HBCD-RNA complexes, no shifting was observed for the band at 269 *nm* (Figure 3) which can be attributed to the presence of a conformation in free RNA and in HBCD-RNA complexes. This is consistent with the infrared results which showed a conformation in free RNA with IR marker bands at 1698 (G), 1241 (PO2), 861 and 810 *cm*^−1^ (ribosephosphate) and in HBCD-RNA complexes at 1689–1688 (G), 1241–1234 (PO2), 864–868 and 812–810 *cm*^−1^ (ribose-phosphate) in the complexes (Figure 4). Besides, minor intensity change of the band at 269 nm and its collapse in the spectrum of HBCD-RNA can be related to the lack of RNA aggregation in the presence of HBCD polymer (Figure 3). This is consistent with DLS results of HBCD-RNA complexes which revealed that RNA aggregation did not occur.

### Stability of HBCD-RNA complex

**Figure 5.**
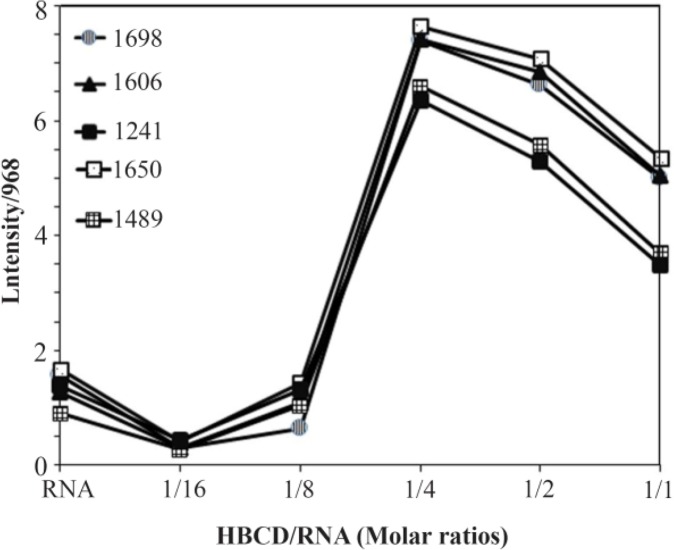
CD spectra at 25°*C* for (a) free RNA (40 *μM* in phosphate buffer, pH=7.4), (b-f) HBCD-RNA complexes with different concentrations of HBCD containing: (b) HBCD (125 *μM*), (c) HBCD (250 *μM*), (d) HBCD (500 *μM*), (e) HBCD (1000 *μM*) and (f) HBCD (2000 *μM*).

HBCD-RNA binding constant was determined as demonstrated previously in UV-Visible spectroscopy (Figure 5A). The overall binding constants were obtained using UV spectroscopy in a previously reported method ^37^. Concentrations of the complexed HBCD (ligand) were determined by subtracting the absorbance of the free RNA at 260 *nm* from those of the complexed. The concentration of free ligand was determined by the subtraction of the complexed ligand from total ligand. The data of 1/[ligand complexed] almost proportionally augmented as a function of 1/[free ligand] (Figure 5B). As the double reciprocal plot of 1/(A−A_0 _) *vs*. 1/(HBCD concentration) was linear, binding constant (K) was calculated from the ratio of the intercept to the slope (Figures 4A and 4B); A_0_ is the initial absorbance of the free RNA at 260 *nm* and A is the recorded absorbance of the complexes at various HBCD concentrations. The overall binding constant of HBCD-RNA (K=1.25×10^3^ *M*^−1^) was indicative of a weak stability through attraction forces between HBCD and RNA. Similar bindings were reported for G_3.0_ PAMAM dendrimers conjugated to 1-pyrene carboxaldehyde G_3.0_-PY (as guest) and HBCD (as host) complexes ^42^ in which the enhanced λ_Max_ of the guest molecule was attributed to the host-guest interactions. Besides, UV-Vis spectrum of RNA at 260 *nm* (Figures 5A and 5B) exhibited increased intensity upon the addition of HBCD (spectra c to j) which was assigned to the host-guest interactions between the two segments and formation of HBCD-RNA complex.

### RNA conformation

RNA remains in a conformation in HBCD-RNA complexes. The lack of major shifting of the A-RNA marker bands at 815–809 *cm*^−1^ (phosphodiester), 867–861 (ribosephosphate), 1247–1241 (phosphate), and 1700–1688 (guanine) indicates that RNA remains in a conformation upon HBCD complexation (Figure 1) ^43–45^.

### Size and zeta potential

The size distribution profile of small particles in suspension or (macro) molecules in solution can be determined by Dynamic Light Scattering (DLS) ^46^. DLS measurements were applied to estimate the average hydrodynamic diameterof RNA, HBCD and their supramolecular assembly in solution (HBCD-RNA complexes) (Figures 6A–C). The obtained hydrodynamic diameter for free HBCD (2.8 *nm* at pH=7.4 and at 37°*C*) is in good agreement with previous literature reports (Figure 6) ^28^. After addition of HBCD to RNA solution, the d_h_ value of the HBCD-RNA complex increased to 5.7 *nm* which is larger than the sum of the hydrodynamic diameters of the pure components. As discussed earlier, by formation of HBCD-RNA complex, RNA remained in a conformation. Therefore, increase in HBCD-RNA dimension can be attributed to an extended conformation of the polyglycerol chains of HBCD into the solvated environment. Based on the interactions between albumin and HBCD, this result could also be explained by the structural properties of HBCD and cyclodextrin hydrophobic cavity, the polar outer shell as well as complex tendency to interact with RNA ^47^. RNA zeta potential due to the presence of ionized residues on RNA surface is greatly affected by the concentration and charge of the cationic particles. Indeed, the phosphate backbone of nuleic acids carries one negative charge per residue ^48^. By the addition of HBCD (ζ=−1.23 *mV*), the value of ζ for the supramolecular complex (HBCD-RNA) decreases to −9.22 *mV*. Decrease in ζ-value can be resulted from the intermolecular forces which led to HBCD-RNA supramolecular assembly.

## Conclusion

An unprecedented type of gene delivery system was successfully developed based on linear dendritic supramolecular biopolymer. RNA-HBCD complexes can be used to gain insight into the new strategies for gene delivery. Our study provided substantial quantitative data on the effect of low and high HBCD concentrations on polynucleotide structure and the binding affinity of RNA to HBCD. According to the spectroscopic results, the following points are considerable: RNA binds HBCD through non-covalent binding (host-guest interactions) between the gene bases as guest and β-cyclodextrine cavities in HBCD as host. The binding constant (K=1.25×10^3^) indicated weak HBCD interaction with RNA which easily dissociates in aqueous solution. Strong bonds between RNA and HBCD were formed at higher HBCD concentrations. The small sized synthesized complex (5 *nm*) successfully prevented RNA aggregation and protected RNA by host-guest interactions between the cavity of HBCD polymers and the bases of gene. No RNA conformational change occurred upon HBCD interaction and RNA remained in A-family structure.
